# Comprehensive systematic review and pooled analysis of real‐world studies evaluating immunomodulator and biologic therapies for chronic pouchitis treatment

**DOI:** 10.1002/jgh3.13000

**Published:** 2023-12-07

**Authors:** Emi Khoo, Andrew Lee, Teresa Neeman, Yoon‐Kyo An, Jakob Begun

**Affiliations:** ^1^ Mater Hospital Brisbane Brisbane Queensland Australia; ^2^ School of Medicine University of Queensland Brisbane Queensland Australia; ^3^ Mater Research Brisbane Queensland Australia; ^4^ Biology Data Science Institute Australian National University Canberra Australian Capital Territory Australia

**Keywords:** biologic therapies, chronic pouchitis, immunomodulator

## Abstract

**Background and Aim:**

Pouchitis is a common complication after restorative ileal pouch–anal anastomosis following proctocolectomy for ulcerative colitis. Antibiotic‐dependent or antibiotic‐refractory chronic pouchitis (CP), which is a common cause of pouch failure affecting 15–20% of patients, is challenging to treat. The efficacy of second‐line immunomodulator and biologic therapy remains poorly defined. We present a pooled analysis of real‐world efficacy data from peer‐reviewed full‐text manuscripts, focusing on immunomodulator and biologic therapies in CP.

**Methods:**

Embase and PubMed databases were searched for full‐text articles describing the treatment of CP. We performed a systematic review and pooled analysis of published studies to assess the efficacy of immunomodulators, including thiopurines and methotrexate, and biologics including antitumor necrosis factor, anti‐integrin, and interleukin‐12/23 antagonists. Clinical and endoscopic response and remission rates were combined for pooled analyses. Rates of treatment discontinuation and safety were also assessed.

**Results:**

Pooled analysis comprised 20 full‐text articles (485 patients). Overall clinical response rate was 46% (95% CI: 35–59%) and clinical remission rate was 35% (95% CI: 21–52%). Overall endoscopic response and remission rates were 41% (95% CI: 18–68%) and 15% (95% CI: 5–39%), respectively. Individual agents' safety profile was reassuring, with vedolizumab being the most favorable.

**Conclusion:**

The real‐world efficacy data of immunomodulators in the treatment of CP is insufficient. Vedolizumab and ustekinumab appeared effective and safe for CP, whereas anti‐TNFs showed higher rates of adverse events. The high heterogeneity within the studies is attributed to the real‐world study design, obfuscating drug efficacy comparisons across the studies. Further studies are required to define the comparative effectiveness of available treatments of CP.

## Graphical abstract

This systematic review and pooled‐analysis evaluated the real‐world efficacy data of immunomodulators and advanced therapies in treatment of chronic pouchitis. Vedolizumab and ustekinumab appeared effective and safe for chronic pouchitis, whereas anti‐TNFs showed higher adverse events rates.

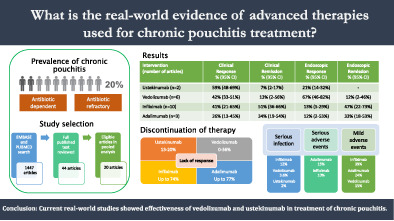

## Introduction

Pouchitis is a common chronic complication of restorative proctocolectomy and ileal pouch–anal anastomosis (IPAA) surgery among patients with inflammatory bowel disease (IBD; medically refractory ulcerative colitis or colitis‐associated neoplasia) or familial adenomatous polyposis (FAP).^1^, ^2^ Pouchitis is a common complication, occurring in up to 23–60% of patients with IBD^3^, but is less common in FAP, affecting 0–11% of individuals. The Crohn's and Colitis Foundation of America Partners cohort reported a higher overall incidence of self‐reported pouchitis in 79% of patients.^4^ Symptoms of pouchitis are debilitating and include frequent diarrhea, haematochezia, abdominal pain, urgency, and fecal incontinence.^5^

First‐line therapy for acute pouchitis is antibiotics, which is highly effective.^1^, ^6^ However, up to 15–20% of patients develop chronic pouchitis (CP).^7^, ^8^, ^9^ CP can be classified as chronic antibiotic‐dependent pouchitis (CADP) or chronic antibiotic‐refractory pouchitis (CARP). CADP is defined as that found in patients who require more than 4 weeks of antibiotics within a 12‐month period to relieve symptoms, and CARP is defined as that found in patients who fail to achieve an adequate response despite a 4‐week course of antibiotics.

Immunomodulator and biologic therapies are often used to manage CP. Evidence supporting their use is largely limited to retrospective cohort studies and pilot clinical trials. There are two published combined systematic reviews and meta‐analyses comparing the efficacy of infliximab, adalimumab, and tacrolimus with conventional therapies.^1^, ^5^ A systematic review on the use of antitumor necrosis factor (anti‐TNF) therapy, comparing the efficacy of infliximab to adalimumab, was also published in 2015.^10^ Newer biologic therapies (ustekinumab and vedolizumab) were not included in those analyses. To investigate the newly available evidence for therapies in CP, we performed a systematic review and pooled analysis to determine the efficacy of immunomodulator and biologic therapies for the treatment of CP. Our study is a comprehensive pooled analysis, encompassing a wide range of real‐world data derived from peer‐reviewed full‐text articles. The primary objective of our study was to examine the effectiveness and outcomes of immunomodulator and biologic therapies, specifically in the context of CP. By synthesizing and analyzing data from various high‐quality sources, our study aims to provide a compressive and in‐depth understanding of the real‐world implications and benefits of these therapeutic interventions in managing CP.

## Materials and methods

This study was conducted in accordance with the Preferred Items for Systematic Reviews and Meta‐analysis (PRISMA) guidelines. Our systematic review was registered with the International prospective register of systematic reviews (PROSPERO) on August 7, 2020 (reference number: CRD42020196627).

### Study selection

A structured search of Embase and PubMed was performed on July 16, 2023, to identify all studies that assessed the efficacy of immunomodulators and/or biologic therapies in CP from inception to June 2023. The Medical Subject Heading (MeSH) terms used were “pouchitis,” “ulcerative colitis,” “therapy,” “immunomodulating agent,” “methotrexate,” “azathioprine,” “mercaptopurine,” “tioguanine,” “thioguanine derivatives,” “tumor necrosis factor inhibitor,” “infliximab,” “adalimumab,” “golimumab,” “vedolizumab,” and “ustekinumab.”

Articles meeting the search criteria were screened by two authors (E.K. and A.L.) and reviewed independently by another two (Y.A. and J.B.). Any discrepancies that arose during the screening process were addressed through discussion till reaching a consensus among the authors. Inclusion criteria were as follows: studies involving adult patients (age >18) who had undergone IPAA for UC; articles that were published in complete form in peer‐reviewed journals; and prospective or retrospective studies including cohort studies and case series of at least three patients treated for chronic pouchitis. We excluded randomized controlled trials, review articles, abstracts, non‐English‐language articles, studies reporting fewer than three cases of chronic pouchitis, and articles with duplicated results.

### Data extraction and outcome measures

The primary outcome was the clinical response rate at 6 months (with a 4‐month window) following induction. Secondary outcomes included clinical remission, endoscopic response and mucosal healing rate at 6 month, and discontinuation rate. Clinical and endoscopic responses were defined as a reduction of clinical and endoscopic sub‐scores of the pouchitis disease activity index (PDAI) by 2 points or more. Clinical remission and mucosal healing were defined as clinical and endoscopic sub‐scores of the PDAI of less than 3. Reasons for discontinuation for each intervention were extracted, which included serious adverse events, surgical intervention, and switch of therapy due to treatment failure.

### Grading of evidence

Risk‐of‐bias assessment was completed by two independent authors (E.K. and A.L.), based on the Risk Of Bias In Non‐randomized Studies of Interventions (ROBINS‐I) tool.^11^ ROBINS‐I addresses pre‐intervention, at‐intervention, and post‐intervention features of the study. It is structured into domains of bias, which include confounding, selection, information, and reporting bias. The judgment of each bias domain and overall risk of bias are classed as “Low,” “Moderate,” “Serious,” or “Critical.” In instances where two reviewers had differing opinions, disagreements were resolved through thorough discussion to achieve consensus.

### Statistical analysis

The overall rates and 95% confidence intervals (CIs) were estimated for clinical response, clinical remission, endoscopic response, and mucosal healing, using a generalized linear mixed model in which the linear predictor contained random effects in addition to the usual fixed effects. Modeling was performed using the *metaprop* function in the *meta* package (version 4.12–0) in R (version 3.6.3). For discontinuation rate, the events including IBD‐related surgery, switch of therapy, and serious adverse events were combined. Statistical heterogeneity described the percentage of total variation across studies attributable to between‐study heterogeneity. This heterogeneity was determined using the *Q*‐statistic of *I* ^2^. Chi‐squared test with a *P*‐value <0.10 or an *I* ^2^‐value >50% indicates substantial heterogeneity.

## Results

### Study selection

**Figure 1:**
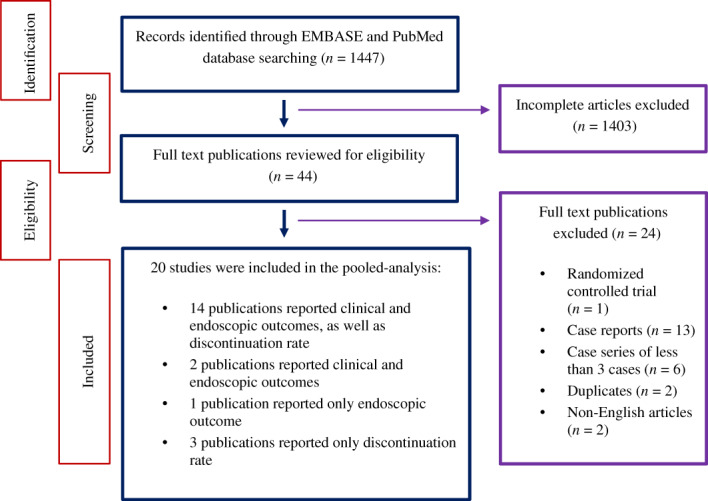
PRISMA flowchart illustrating the search process.

Literature search in Embase and PubMed identified a total of 1447 publications. After initial screening based on paper titles and abstracts, 44 full‐text articles were reviewed. Twenty articles were considered eligible for pooled analysis following the application of inclusion and exclusion criteria (Fig. 1).

### Grading of evidence

Ten studies were graded as “Serious” risk of bias, and 10 were graded as “Critical” (Table 1). Bias due to confounding and measurement of outcomes were the two main domains of concern.

**Table 1:** ROBINS‐I bias assessment of the studies

Study	Confounding	Selection	Classification of intervention	Deviation from intended intervention	Missing data	Measurement of outcome	Reporting of outcomes	Overall risk of bias
Ollech *et al*.^12^								
Weaver *et al*.^13^								
Gregory *et al*.^14^								
Singh *et al*.^15^								
Verstockt *et al*.^16^								
Bar *et al*.^17^								
Khan *et al*.^18^								
Philpott *et al*.^19^								
Segal *et al*.^20^								
Kelly *et al*.^21^								
Uchino *et al*.^22^								
Viazis *et al*.^23^								
Barreiro‐De Acosta *et al*.^24^								
Haveran *et al*.^25^								
Ferrante *et al*.^26^								
Calabrese *et al*.^27^								
Viscido *et al*.^28^								
Shen *et al*.^29^								
Barreiro‐De Acosta *et al*.^24^								
Uchino *et al*.^30^								

LOW	MODERATE	SERIOUS	CRITICAL
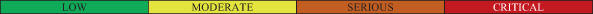			

### Study characteristics

A total of 20 studies were included in this analysis, which included 10 retrospective single‐arm cohort studies, 2 retrospective comparative cohort studies, 1 case series (*n* = 4), and 7 pilot studies. The two retrospective comparative cohort studies included three arms: one study compared infliximab, adalimumab, and vedolizumab, and the other study compared infliximab, thiopurine, and a combination of infliximab and thiopurine.

Twelve of the 20 studies defined CP at baseline as the prolonged use of antibiotics for 4 weeks or more, while the remainder defined it as ongoing symptoms despite antibiotic use. Endoscopic confirmation of pouchitis at baseline was performed in 15 studies. Interventions included in the pooled analysis were ustekinumab (2 articles), vedolizumab (6 articles), infliximab (10 articles), adalimumab (3 articles), tacrolimus enema (1 article), thiopurine (1 article), and combination therapy of infliximab and thiopurine (1 article). All therapies were administered using standard dosing regimens for ulcerative colitis.

A total of 495 patients from these studies were pooled for analysis. The two largest interventional cohorts were infliximab (*n* = 195) and vedolizumab (*n* = 153) (Table 2). Nearly 50% (*n* = 241) of patients were female, with age ranging from 22 to 54 years. The reported time since pouch surgery at commencement of intervention ranged from 1 to 10 years, based on available data reported in 13 studies. The mean follow‐up period ranged from 2 to 12 months.

**Table 2:** Characteristics of the studies

Study	Types of study	Intervention	Total cases	Median follow‐up (month)	Clinical response *n* (%)	Clinical remission *n* (%)	Endoscopic response *n* (%)	Endoscopic remission *n* (%)
Ollech *et al*.^12^	Retrospective	Ustekinumab	24	13	12 (50%)	‐	9 (38%)	0 (0%)
Weaver *et al*.^13^	Retrospective	Ustekinumab	56	12	35 (62.5%)	40 (7%)	34 (60%)	0 (0%)
Gregory *et al*.^14^	Retrospective	Vedolizumab	83	15.5	34 (41%)	14 (17%)	46 (55%)	12 (14%)
Singh *et al*.^15^	Retrospective	Vedolizumab	19	3	6 (2%)	0 (0%)	14 (74%)	‐
Verstockt *et al*.^16^	Retrospective	Vedolizumab	15	14.5	‐	9 (60%)	‐	‐
Bar *et al*.^17^	Retrospective	Vedolizumab	20	3.5	‐	9 (45%)	‐	13 (64%)
Khan *et al*.^31^	Retrospective	Vedolizumab	12	6	8 (67%)	0 (0%)	10 (83%)	0 (0%)
Philpott *et al*.^19^	Retrospective	Vedolizumab	4	4	‐	‐	3 (75%)	0 (0%)
Verstockt *et al*.^16^	Retrospective	Infliximab	23	14.5	‐	10 (44%)	‐	‐
Segal *et al*.^20^	Retrospective	Infliximab	34	10	‐	‐	‐	‐
Kelly *et al*.^21^	Retrospective	Infliximab	42	24	31 (74%)	20 (48%)	‐	19 (45%)
Uchino *et al*.^22^	Pilot	Infliximab	10	2	‐	‐	‐	‐
Viazis *et al*.^23^	Pilot	Infliximab	7	12	1 (14%)	5 (71%)	‐	5 (71%)
Barreiro‐De Acosta *et al*.^24^	Retrospective	Infliximab	33	12	11 (33%)	11 (33%)	‐	13 (40%)
Haveran *et al*.^25^	Retrospective	Infliximab	4	38	‐	‐	‐	‐
Ferrante *et al*.^26^	Pilot	Infliximab	25	21	14 (56%)	8 (32%)	4 (15%)	15 (62%)
Calabrese *et al*.^27^	Pilot	Infliximab	10	6	‐	8 (80%)	‐	8 (80%)
Viscido *et al*.^28^	Pilot	Infliximab	7	35	1 (14%)	6 (86%)	2 (29%)	5 (71%)
Shen *et al*.^29^	Pilot	Adalimumab	17	2	4 (24%)	8 (47%)	4 (24%)	7 (41%)
Verstockt *et al*.^16^	Retrospective	Adalimumab	13	14.5	‐	5 (39%)	‐	‐
Barreiro‐De Acosta *et al*.^24^	Retrospective	Adalimumab	10	12	3 (30%)	1 (10%)	0 (0%)	4 (40%)
Uchino *et al*.^30^	Pilot	Tacrolimus	10	2	9 (90%)	7 (70%)	10 (100%)	0 (0%)
Haveran *et al*.^25^	Retrospective	Thiopurine	8	38	‐	‐	‐	‐
Haveran *et al*.^25^	Retrospective	Infliximab + thiopurine	9	38	‐	‐	‐	‐

### Primary outcome

#### Clinical response

**Figure 2:**
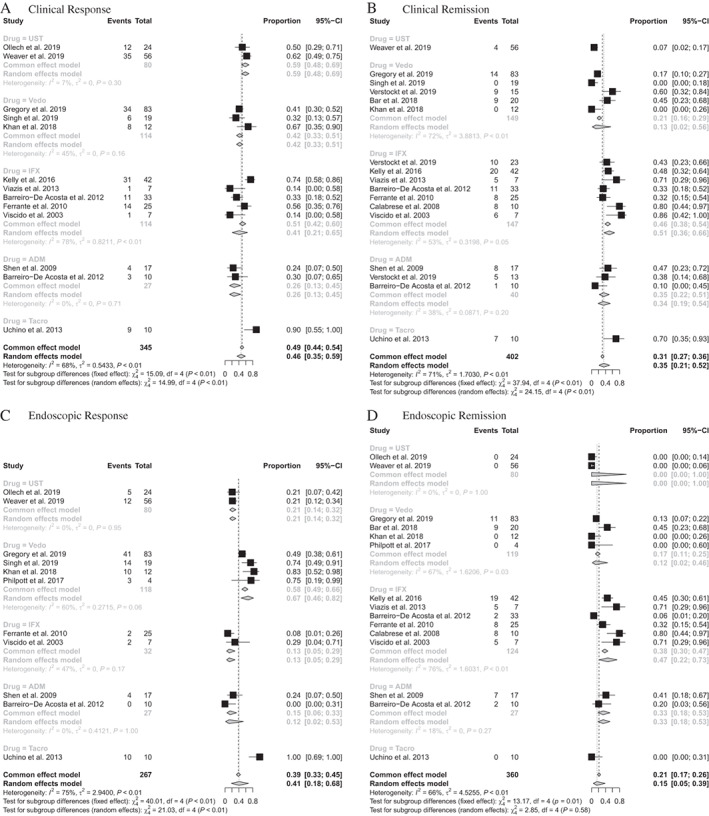
Forrest plots of the primary and secondary outcomes comparing the efficacy of each intervention: (a) clinical response, (b) clinical remission, (c) endoscopic response, and (d) endoscopic remission.

Clinical response was assessed in 13 studies (Table 2 and Fig. 2a), with those treated with infliximab and vedolizumab making up the two largest proportions of the sample. The overall pooled clinical response rate was 46% (95% CI: 35–59%). There was statistically significant between‐study heterogeneity, with an *I* ^2^ value of 68% (*P* < 0.01). The clinical response rate in patients receiving infliximab was 41% (95% CI: 21–65%, *n* = 47); vedolizumab was 42% (95% CI: 33–51%, *n* = 48), adalimumab was 26% (95% CI: 13–45%, *n* = 7), ustekinumab was 59% (95% CI: 48–69%, *n* = 47), and tacrolimus enemas was 90% (95% CI: 55–100%, *n* = 9).

### Secondary outcomes

#### Clinical remission

Seventeen studies, comprising 402 patients, reported on rates of clinical remission (Table 2, Fig. 2b). The majority of patients received infliximab (*n* = 147) or vedolizumab (*n* = 149). The pooled clinical remission rate for immunomodulator and biologic therapies was 35% (95% CI: 21–52%) with a high degree of between‐study heterogeneity (*I* ^2^ = 71%, *P* < 0.01). Clinical remission was observed in 51% of patients treated with infliximab (95% CI: 36–66%, *n* = 75), in 13% with vedolizumab (95% CI: 2–56%, *n* = 19), in 34% with adalimumab (95% CI: 19–54%, *n* = 14), in 7% with ustekinumab (95% CI: 2–17%, *n* = 4) and in 70% with tacrolimus (95% CI: 35–93%, *n* = 7).

#### Endoscopic response

Eleven studies evaluated the endoscopic response (Table 2 and Fig. 2c). The overall endoscopic response rate was 41% (95% CI: 18–68%). There was significant between‐study heterogeneity with an *I* ^2^ value of 75% (*P* < 0.01). Patients receiving vedolizumab accounted for almost half (44%) of the pooled cohort, with an individual endoscopic response rate of 67% (95% CI: 46–82%). The other endoscopic response rates were 21% for ustekinumab (95% CI: 14–32%, *n* = 17), 13% for infliximab (95% CI: 5–29%, *n* = 4), 12% for adalimumab (95% CI: 2–53%, *n* = 4), and 100% for tacrolimus enemas (95% CI: 69–100%, *n* = 10).

#### Endoscopic remission

Fifteen studies reported on endoscopic remission (Table 2, Fig. 2d). The combined results demonstrated a pooled endoscopic remission rate of 15% (95% CI: 5–39%) with significant heterogeneity, with an *I* ^2^ value of 66% (*P* < 0.01). Endoscopic remission was seen in 47% of patients treated with infliximab (95% CI: 22–73%, *n* = 47), in 12% with vedolizumab (95% CI: 2–46%, *n* = 20), in 33% with adalimumab (95% CI: 18–53%, *n* = 9), and in 0% with ustekinumab and tacrolimus.

#### Discontinuation of therapy

Sixteen studies documented the rate of discontinuation at the end of the follow‐up period, which ranged from 2 to 38 months (Table 3). Five studies had a follow‐up period of 6 months or less, one study reported a 10‐month follow‐up period, and the remainder reported follow‐up data at 12 month or beyond.

**Table 3:** Reasons for discontinuation of therapy

Study	Intervention	Total cases	Median follow‐up (month)	Discontinuation *n* (%)	Total adverse events *n* (%)
Ollech *et al*.^12^	Ustekinumab	24	13	5 (20%)	‐
Weaver *et al*.^13^	Ustekinumab	56	12	7 (13%)	1 (2%)
Gregory *et al*.^14^	Vedolizumab	83	15.5	30 (36%)	5 (6%)
Singh *et al*.^15^	Vedolizumab	19	2	4 (21%)	2 (11%)
Verstockt *et al*.^16^	Vedolizumab	15	14.5	4 (27%)	0 (0%)
Bar *et al*.^17^	Vedolizumab	20	3.5	0 (0%)	3 (15%)
Verstockt *et al*.^16^	Infliximab	23	14.5	17 (74%)	9 (39%)
Segal *et al*.^20^	Infliximab	34	10	18 (53%)	4 (12%)
Kelly *et al*.^21^	Infliximab	42	24	16 (38%)	5 (12%)
Uchino *et al*.^22^	Infliximab	10	2	3 (30%)	‐
Viazis *et al*.^23^	Infliximab	7	12	1 (14%)	1 (14%)
Barreiro‐De Acosta *et al*.^24^	Infliximab	33	12	13 (39%)	5 (21%)
Haveran *et al*.^25^	Infliximab	4	38	1 (25%)	0 (0%)
Ferrante *et al*.^26^	Infliximab	25	21	12 (48%)	2 (8%)
Viscido *et al*.^28^	Infliximab	7	35	0 (0%)	1 (14%)
Shen *et al*.^29^	Adalimumab	17	2	3 (18%)	4 (24%)
Verstockt *et al*.^16^	Adalimumab	13	14.5	10 (77%)	2 (15%)
Barreiro‐De Acosta *et al*.^24^	Adalimumab	10	12	4 (40%)	‐
Uchino *et al*.^30^	Tacrolimus	10	2	0 (0%)	3 (30%)
Haveran *et al*.^25^	Thiopurine	8	38	1 (13%)	1 (13%)
Haveran *et al*.^25^	Infliximab + thiopurine	9	38	5 (56%)	0 (0%)

The main reason for discontinuation was lack of response. The highest rate was reported in cohorts receiving adalimumab (up to 77%), followed by infliximab (up to 74%) and combination therapy with infliximab and thiopurine (56%). Ustekinumab, vedolizumab, tacrolimus, and thiopurine cohorts reported lower discontinuation rates of 13–20%, 0–36%, 0%, and 13%, respectively.

The need for surgical interventions, including end ileostomy and anastomotic stricture dilatation, was observed most frequently in the cohort treated with combination infliximab and thiopurine therapy (56%). Other biologic monotherapies (adalimumab, infliximab, vedolizumab, and ustekinumab) were found to have similar surgical interventional rates, in the range 15–40%, 0–30%, 0–46%, and 20%, respectively.

Adverse events were reported in 75% of the studies. Serious infections, including *Clostridium difficile* infection, sepsis, intra‐abdominal abscess, and shingles were reported with ustekinumab (2%), vedolizumab (3.6%), and infliximab (12%). Serious adverse events, in particular anaphylaxis and delayed hypersensitivity reaction, were observed only with infliximab (up to 10%) and adalimumab (15%). Pancreatitis was documented in one patient treated with a thiopurine. Mild adverse events, including headache, injection site reaction, nausea, and rash, were most common in the infliximab intervention group, with the highest reported rate of 39%, followed by adalimumab (24%). Thirty percent of the tacrolimus enema cohort reported mild burning in the pouch upon application. Low rates of mild adverse events were noted in the vedolizumab cohort (up to 15%), but none was reported in the ustekinumab cohort.

## Discussion

Pouchitis frequently occurs after IPAA surgery and presents complex management challenges. Although antibiotics are effective for treating acute pouchitis,^1^ up to 20% of patients develop CP, including CADP or CARP. Second‐line therapies such as steroids, bismuth, elemental diet, fecal microbiota transplant, probiotics, glutamine suppository, butyrate suppository, and adalimumab are not reported as achieving significant rates of clinical remission.^1^, ^5^ There is an urgent need to develop effective treatments for CP given its significant burden on physical health and quality of life and also the significant associated economic burden.^9^

The pathogenesis of pouchitis remains uncertain. Responsiveness to antibiotic therapy indicates the microbial‐driven etiology of acute pouchitis.^32^ Many studies have suggested that the development of CP is multifactorial, similar to IBD itself. The proposed etiologies include bacterial dysbiosis within the ileo‐anal pouch, immune dysregulation, mucosal ischemia and oxygen‐free radical injury, reduction in short‐chain fatty acids, and genetic predisposition.^33^ Histologically, the ileal pouch mucosa shows colonic metaplasia with increased crypt cell proliferation and chronic inflammatory cell infiltrate within the lamina propria.^33^ Given that the histological features are analogous to those of ulcerative colitis, it is plausible that immunomodulators and biologic therapies would be beneficial in the setting of CP. The meta‐analysis by Chandan *et al*. (2021) demonstrated the effectiveness of biologic therapy in chronic pouchitis; however, the efficacy of immunomodulators was not assessed.^34^ To date, our study represents the most extensive pooled analysis of real‐world data sourced from peer‐reviewed full‐text articles focusing on immunomodulator and biologic therapies in CP. By synthesizing and analyzing a diverse range of high‐quality sources, our study offers a comprehensive and profound exploration of the practical implications and advantages of these therapeutic interventions in managing CP.

A single‐cohort study of 10 patients receiving tacrolimus enemas was included in this pooled analysis. The clinical response (90%), clinical remission (70%), and endoscopic response (100%) rates were found to be the highest among all interventions. The results of this study must be interpreted with caution, however, because of the small sample size and selection bias. Endoscopic remission was achieved in 5% of patients, suggesting that topical tacrolimus may be useful in inducing short‐term clinical response but not mucosal healing. Although none of the patients had discontinued therapy at the end of the follow‐up period, 30% reported mild burning and no patient was able to tolerate the tacrolimus enema for 10 min. No serious adverse events were documented, supporting the relative safety of local tacrolimus application.

Thiopurine use in CP was reported in a retrospective study of eight patients, which focused on the safety profile of therapy. After 38 months of follow‐up, one patient discontinued therapy due to acute pancreatitis. Thiopurine‐induced pancreatitis is a well‐recognized complication occurring in approximately 3% of patients treated with thiopurines.^17^

In our pooled analysis, the largest cohort of patients was treated with infliximab, and this treatment was associated with high clinical and endoscopic remission rates of 51% (95% CI: 36–66%; *I* ^2^ = 58%; seven studies; 58/147 patients) and 47% (95% CI: 22–73%; *I* ^2^ = 76%; six studies; 47/124 patients), respectively. These remission rates are in general agreement with published data by Huguet *et al*. (2018).^35^ Clinical response was reported in a number of studies and was achieved in 41% (95% CI: 21–65%; *I* ^2^ = 78%; five studies; 47/114 patients) of patients; however, there was significant heterogeneity between studies. The reported endoscopic response rate was low at 13% (95% CI: 5–29%; *I* ^2^ = 47%; two studies; 4/32 patients). The contrast in rates of clinical and endoscopic response compared to remission may reflect differences in available data, as there was significant variability in outcomes reported among studies. The discontinuation rate in the infliximab cohort was noted to be as high as 74% in one of the retrospective studies.^15^ Reasons for discontinuation included lack or loss of response and adverse events. Up to 30% of patients proceeded to the formation of a permanent end‐ileostomy. Almost 10% of patients suffered serious adverse events, including anaphylaxis, delayed hypersensitivity, and drug‐induced lupus. In addition, up to 12% of patients had severe infections, including sepsis and shingles. Hence, the rate of adverse events must be considered in patients receiving infliximab for CARP or CADP.

Similarly, the use of adalimumab in CP was associated with high rates of discontinuation and progression to surgical management—up to 77% and 40%, respectively. Adverse events included delayed hypersensitivity (15%), headache, and injection‐site reaction (24%), but no serious infections were reported. The safety profile for adalimumab was similar to previously published IBD data.^36^ Overall, clinical and endoscopic remission rates with adalimumab were 34% and 33%, respectively, with low between‐study heterogeneity. The adalimumab cohort had a low clinical response rate of 26% (95% CI: 13–45%). This may be due to differences in the patient populations reported, particularly the inclusion of patients who had previously failed infliximab in studies investigating adalimumab. One small randomized controlled trial of adalimumab identified using our database search strategy was not included in the pooled analysis.^18^ This small study showed no significant difference in response between the placebo and adalimumab arms. Taken together, these results indicate the marginal efficacy of adalimumab for the treatment of CP.

There were six retrospective studies assessing the efficacy of vedolizumab in CP, including patients in whom anti‐TNF therapy had failed previously. Clinical and endoscopic response rates were 42% and 67%, respectively, without significant between‐study heterogeneity. However, the clinical and endoscopic remission rates were poor, 13% and 12%, respectively, with substantial between‐study heterogeneity. The rates of discontinuation and surgical management were both up to 36%, which is lower than that obseved with infliximab or adalimumab. The safety profile of vedolizumab was reassuring, with only three cases of serious infection (6%), which included *C. difficile* infection and intra‐abdominal sepsis. No other serious adverse events were reported. Vedolizumab appears to achieve clinical and endoscopic response with a good safety profile similar to that seen in IBD studies.^35^ The efficacy of vedolizumab was recently demonstrated in the EARNEST trial published in *New England Journal of Medicine* in March 2023 by Travis *et al*., a well‐powered, double‐blind, randomized, placebo‐controlled trial for the treatment of CP.^37^ Among the 102 patients randomized, the clinical and endoscopic remission rate was 31%, compared to 10% in the placebo group, at the end of induction (95% CI: 5–38%, *P* = 0.01). Serious adverse events were seen in 6% of the vedolizumab group, which is similar to the findings in our pooled analysis.

Ustekinumab was recently approved for use in IBD, and there have been two published retrospective studies assessing the efficacy of ustekinumab in CP. A large proportion of patients (70%) had tried anti‐TNF prior to ustekinumab treatment. The clinical response rate was high at 59% (95% CI: 48–69%), with negligible between‐study heterogeneity. However, clinical remission, endoscopic response, and healing rates were low—7%, 21%, and 0%, respectively. Compared to the published data on the efficacy of induction with ustekinumab in ulcerative colitis, the clinical response rate in CP was similar but with a lower endoscopic improvement rate.^38^, ^39^ The rate of discontinuation was up to 20% due to loss of response, while 8% progressed to a permanent end‐ileostomy. There was a single case of *C. difficile* infection but no other serious adverse events were reported. These data suggest that ustekinumab is safe and able to achieve clinical and endoscopic responses, but not necessarily remission, during the follow‐up period as reported in the published studies.

This analysis provides an up‐to‐date and comprehensive assessment of the available studies examining immunomodulator and biologic therapy for the treatment of CP. Our focus on real‐world studies reflects the routine clinical practice and patient populations encountered in everyday healthcare settings. Therefore, the findings have high external validity, and are likely to be applicable to a broad range of patients and healthcare settings. This real‐world study captures the actual treatment patterns and variations in clinical practice, reflecting the choices made by healthcare providers and patients. This information is crucial for understanding the impact of interventions in routine care and evaluating their effectiveness in a real‐world context. Although real‐world studies have these strengths, it is important to acknowledge their limitations, such as potential biases, confounding factors, and the absence of randomization. There were 20 studies with high between‐study heterogeneity that were included in the pooled analysis. Most studies were single‐centered cohort studies with small sample sizes. The lack of high‐quality head‐to‐head trials and the heterogeneity of the patient populations studied make it challenging to compare the benefits of one drug with those of another, and conclusions about the comparative efficacy of each agent cannot be drawn from the available data. For these reasons, the data presented should be interpreted with caution. Through our analysis, we also found that inconsistent definitions of disease activity were used, which contribute to considerable between‐study heterogeneity, affecting the interpretation and restricting the extrapolation of some findings to clinical practice. To overcome these limitations, large‐scale, multicenter, randomized, placebo‐controlled trials using consensus definitions of pouchitis activity and standardized outcome measures are required. These real‐world studies play a crucial role in complementing the findings of controlled trials and generating evidence that better reflects the complexities of real‐world healthcare settings. Conducting a randomized controlled trial can be challenging for many reasons, including ethical considerations, cost and resource intensiveness, patient recruitment and retention, study compliance and patient adherence, as well as practical and generalizability limitations. However, it is considered the gold standard in research design to provide rigorous evidence for the effectiveness and safety of interventions, leading to more reliable and trustworthy results that can inform evidence‐based practice and decision making in healthcare. Hence, careful design of future studies to achieve this standard is warranted.

## Conclusion

Treatment of CADP and CARP remains challenging because of a lack of evidence guiding therapeutic decision making. To date, there is only one published, well‐powered, and randomized controlled trial demonstrating the efficacy of vedolizumab in inducing remission in patients with CP. The current review and pooled analysis of real‐world studies also identified the effectiveness of ustekinumab in achieving clinical response in CP, with a reassuring safety profile. Infliximab was found to be effective in inducing clinical and endoscopic remission, but there were significant rates of treatment failure and adverse events. Adalimumab demonstrated high rates of treatment discontinuation and progression to surgical outcomes with low clinical and endoscopic remission rates. There are limited published data regarding the use of immunomodulators for CP, so no conclusion can be drawn. Further studies are required, but given the efficacy and safety data summarized in this study, current evidence favors the use of vedolizumab and ustekinumab for the treatment of CADP or CARP.
